# *Trichoderma asperelloides* PSU-P1 Induced Expression of Pathogenesis-Related Protein Genes against Gummy Stem Blight of Muskmelon (*Cucumis melo*) in Field Evaluation

**DOI:** 10.3390/jof8020156

**Published:** 2022-02-04

**Authors:** Warin Intana, Prisana Wonglom, Nakarin Suwannarach, Anurag Sunpapao

**Affiliations:** 1 School of Agricultural Technology and Food Industry, Walailak University, Nakhon Si Thammarat 80161, Thailand; iwarin@wu.ac.th; 2 Faculty of Technology and Community Development, Phatthalung Campus, Thaksin University, Songkhla 93110, Thailand; prisana.w@tsu.ac.th; 3 Research Center of Microbial Diversity and Sustainable Utilization, Chiang Mai University, Chiang Mai 50200, Thailand; suwan.462@gmail.com; 4 Agricultural Innovation and Management Division (Pest Management), Faculty of Natural Resources, Prince of Songkla University, Songkhla 90110, Thailand; 5 Center of Excellence on Agricultural Biotechnology (AG-BIO/MHESI), Bangkok 10900, Thailand

**Keywords:** BCAs, disease resistance, gene expression, chitinase, β-1,3-glucanase

## Abstract

Gummy stem blight caused by *Stagonosporopsis cucurbitacearum* is the most destructive disease of muskmelon cultivation. This study aimed to induce disease resistance against gummy stem blight in muskmelon by *Trichoderma asperelloides* PSU-P1. This study was arranged into two crops. Spore suspension at a concentration of 1 × 10^6^ spores/mL of *T. asperelloides* PSU-P1 was applied to muskmelon to investigate gene expression. The expression of PR genes including chitinase (*chi*) and β-1,3-glucanase (*glu*) were determined by reverse transcription quantitative polymerase chain reaction (RT-qPCR), and enzyme activity was assayed by the DNS method. The effects of *T. asperelloides* PSU-P1 on growth, yield, and postharvest quality of muskmelon fruit were measured. A spore suspension at a concentration of 1 × 10^6^ spore/mL of *T. asperelloides* PSU-P1 and *S. cucurbitacearum* was applied to muskmelons to determine the reduction in disease severity. The results showed that the expression of *chi* and *glu* genes in *T. asperelloides* PSU-P1-treated muskmelon plants was 7–10-fold higher than that of the control. The enzyme activities of chitinase and β-1,3-glucanase were 0.15–0.284 and 0.343–0.681 U/mL, respectively, which were higher than those of the control (pathogen alone). Scanning electron microscopy revealed crude metabolites extracted from *T. asperelloides* PSU-P1-treated muskmelon plants caused wilting and lysis of *S. cucurbitacearum* hyphae, confirming the activity of cell-wall-degrading enzymes (CWDEs). Application of *T. asperelloides* PSU-P1 increased fruit weight and fruit width; sweetness and fruit texture were not significantly different among treated muskmelons. Application of *T. asperelloides* PSU-P1 reduced the disease severity scale of gummy stem blight to 1.10 in both crops, which was significantly lower than that of the control (2.90 and 3.40, respectively). These results revealed that application of *T. asperelloides* PSU-P1 reduced disease severity against gummy stem blight by overexpressed PR genes and elevated enzyme activity in muskmelon plants.

## 1. Introduction

Gummy stem blight is a devastating disease of muskmelon cultivation. The disease is caused by the fungal pathogen *Stagonosporopsis cucurbitacearum* [1,2]. Interactions between the plant and fungi trigger a defense response in the plant, which is associated with disease resistance [3]. Plants have developed an elaborate defense response to combat such stresses [4,5]. Plants are also capable of producing pathogenesis-related (PR) protein to defend themselves from infecting fungi [6,7]. The PR proteins, PR2 (β-1,3-glucanase) and PR3 (chitinase), produced by plants are considered the main key enzymes responsible for degrading fungal cell walls, resulting in resistance against plant diseases [8,9]. β-1,3-Glucanase and chitinase hydrolyze the fungal cell wall components β-glucan and chitin, respectively, into small molecules [10,11]. Plants that contain high activity of β-1,3-glucanase and chitinase are able to resist plant diseases [12].

*Trichoderma* species are often collected and isolated from the soil rhizosphere and soil habitat [13]. *Trichoderma* species are known as good biological control agents (BCAs) to control fungal diseases with multifaceted mechanisms [14]. They are strong antagonists due to their capacity to compete for nutrients and space [15], produce antifungal metabolites [13,16], induce the defense response [17], and increase plant growth [18]. Some studies have shown that *Trichoderma* species can produce cell-wall-degrading enzymes (CWDEs) and release them into cell-free culture filtrate, which restricts fungal growth [19]. Furthermore, *Trichoderma asperellum* T1 can induce the defense response by elevating β-1,3-glucanase and chitinase enzyme activity against leaf spot in lettuce [5]. Therefore, the ability to produce CWDEs by antagonistic microorganisms and the ability to induce plants to produce those enzymes using a BCA may be of high interest for managing plant disease.

Our previous research found that *T. asperelloides* PSU-P1 acts as a biological control agent (BCA) with strong antifungal ability against *S. cucurbitacearum*, the pathogen of gummy stem blight, and can reduce disease severity at the seedling stage [20]. However, the plant response to the BCA with long-term use throughout the plant’s lifespan has not been clarified. Altogether, the ability of BCA to induce disease resistance in muskmelon in mature plants, to the harvesting stage, against gummy stem blight disease has also not been determined. Therefore, this research aimed to investigate the effect of *T. asperelloides* PSU-P1 on the expression of pathogenesis-related (PR) protein genes, the activity of cell-wall-degrading enzymes, and the postharvest quality of muskmelon.

## 2. Materials and Methods

### 2.1. Sources of Trichoderma and Pathogens

*Trichoderma asperelloides* PSU-P1 was screened for antifungal ability against *S*. *cucurbitacearum* in a previous study [20] and was used as a BCA for biotic stress in this study. Both *T. asperelloides* PSU-P1 and the fungal pathogen *S. cucurbitacearum* were obtained from the Culture Collection of Pest Management Division, Faculty of Natural Resources, Prince of Songkla University, Thailand. *T. asperelloides* PSU-P1 and *S. cucurbitacearum* were cultured on potato dextrose agar (PDA) and incubated at an ambient temperature of 28 ± 2 °C for 5 days before use in this study.

### 2.2. Plant Inoculation

Field trials were conducted to test the effect of *Trichoderma* on the induction of PR genes and CWDE activity. The experiment was set up in two crops: the first crop was cultivated from June to July 2020 and the second crop was cultivated from August to September 2020. Muskmelon plants were cultivated in a polyhouse with 28 ± 2 °C and 12/12 h light/dark cycle. A total of 20 muskmelon plants were grown in sterile soil for 14 days and then subjected to inoculation with *T. asperelloides* PSU-P1. A spore suspension of *T. asperelloides* PSU-P1 was prepared with distilled water (DW) and the concentration was adjusted to 1 × 10^6^ spore/mL. The experiment was conducted by complete randomized design (CRD) with 10 replications (10 plants). The experiment included two treatments: DW, and drenching with *T. asperelloides* PSU-P1 alone. A total of 50 mL of *T. asperelloides* PSU-P1 spore suspension was applied to each muskmelon plant once a week, and young leaves of muskmelon plants were collected at 7, 14, 21, 28, 35, 42, and 49 days post-inoculation. *T. asperelloides* PSU-P1-treated muskmelon plants and those in the control group were subjected to RNA extraction and protein extraction for further study.

### 2.3. RNA Extraction and Quantitative RT-PCR

Young muskmelon leaves were harvested and subjected to RNA extraction by TriZol reagent (ThermoFisher, Waltham, MA, USA) according to the manufacturer’s instructions. A total of 0.1 g of young muskmelon leaves were ground by small mortar and pestle and added to TriZol reagent. After centrifugation (14,000× *g*), supernatants were collected and precipitated with absolute ethanol. Total extracted RNA was air-dried and dissolved in RNase-free DW. The concentration of RNA was measured and approximately 1 ng of total RNA was subjected to reverse transcription to single-strand cDNA followed by reverse transcription quantitative PCR (RT-qPCR) as previously described by Dumhai et al. [21]. The reaction mixture was analyzed using iScript One-Step RT-PCR reagent with SYBR Green (Bio-Rad, Hercules, CA, USA) and 1 ng of RNA templates. Primer pairs used for the amplification of chitinase (*chi*), β-1,3-glucanase (*glu*), and actin (*act*) genes are shown in Table 1. The actin gene was used as an internal reference gene to normalize the variation in input total cDNA templates between the control and *Trichoderma*-treated samples. Relative gene expression was subjected to analysis of fold change in expression relative to actin as a control by Bio-Rad CFX Manager analysis software (Bio-Rad, Hercules, CA, USA).

### 2.4. Protein Extraction and Enzyme Assays

Muskmelon leaf samples (10 g) were ground using a small mortar and pestle in 25 mL of phosphate buffer at pH 7.0 for chitinase and β-1,3-glucanase assay. The homogenates were centrifuged at 14,000× *g* for 10 min at 4 °C, and supernatants were selected and subjected to enzyme assay immediately. The activity of chitinase and β-1,3-glucanase was determined by the 3,5-dinitrosalicylic acid (DNS) method [22]. For chitinase activity, the reaction mixture contained 250 µL of 1% (*w*/*v*) colloidal chitin in 50 mM KPB at pH 7.0 and 250 µL of crude enzyme. For β-1,3-glucanase activity, the reaction mixture contained 250 µL of 1% (*w*/*v*) laminarin in 50 mM acetate buffer at pH 5.5 and 250 µL of crude enzyme. The reaction mixtures were incubated at 50 °C for 30 min. The increase in reducing sugar products was measured using a UV–vis spectrophotometer (UV5300, METASH, Shanghai, China) at 575 nm and 550 nm for chitinase and β-1,3-glucanase, respectively. Enzyme activity was converted to units per milliliter (U/mL).

### 2.5. Scanning Electron Microscopy

If the crude metabolites extracted from *T. asperelloides* PSU-P1 contained CWDEs, incubating fungal mycelia with the crude metabolites could cause changes in hyphal morphology. To observe the morphology change of *S. cucurbitacearum*, scanning electron microscopy was conducted. Crude metabolites were prepared as described in Section 2.4. Agar plugs of *S. cucurbitacearum* cut from 7-day-old colonies were incubated with the crude metabolites of *T. asperelloides* PSU-P1-treated muskmelon plants at 37 °C for 1 h, whereas agar plugs of *T. asperelloides* PSU-P1 incubated in crude metabolites of uninoculated muskmelon served as control. Agar plugs were then fixed in 3% glutaraldehyde at 4 °C for 24 h. Agar plugs were dehydrated in an alcohol series of 30, 50, 60, 70, 80, 90, and 100%. The samples were dried with a critical point dryer (CPD) and sputter-coated with gold. The samples were observed by JSM-580 LV SEM (JEOL, Peabody, MA, USA) at the Scientific Equipment Center, Prince of Songkla University.

### 2.6. Effect of T. asperelloides PSU-P1 on Plant Growth and Postharvest Quality

The growth of muskmelon plants, yield, and quality of fruit were assessed on harvesting day. Stem diameter, shoot length, stem fresh weight of muskmelon plants, fruit weight, and fruit width, were measured. For postharvest quality, sweetness and fruit firmness were analyzed. Fruit firmness (stiffness) was measured by a System TA.XTplus Texture Analyzer (Stable Micro Systems, Godalming, UK) and expressed as bar. The total soluble solids of muskmelons were analyzed using a hand refractometer (N1; Atago Co., Ltd., Tokyo, Japan) and expressed in degrees Brix (°Brix) in muskmelon fruits.

### 2.7. Plant Inoculation and Disease Assessment

To test the effect of *Trichoderma* on the reduction of gummy stem blight disease, field trials were conducted. The experiment was set up in two crops as described in Section 2.2. A total of 40 muskmelon plants were grown in sterile soil for 14 days and then subjected to inoculation with *T. asperelloides* PSU-P1 or *S. cucurbitacearum*. Spore suspension of *T. asperelloides* PSU-P1and *S. cucurbitacearum* was prepared with DW and adjusted to a concentration of 1 × 10^6^ spore/mL. The experiment was conducted by CRD with 10 replications (10 plants). The experiment was composed of four treatments: DW, drenching with *T. asperelloides* PSU-P1 alone, drenching with *S. cucurbitacearum* alone, and drenching with *T. asperelloides* PSU-P1 and challenging with *S. cucurbitacearum*. A total of 50 mL of *T. asperelloides* PSU-P1 and *S. cucurbitacearum* spore suspension was applied into each muskmelon plant once a week until harvesting day. Qualitative disease severity scale was determined on harvesting day by the method previously described by Sunpapao et al. [23], with some modifications, based on assessing the external symptoms of muskmelon plants (0 = no symptom, 1 = small brown symptom <1 cm, 2 = brown symptom >1 cm, 3 = brown symptom with gummy exudate, 4 = stem rot and collapse).

### 2.8. Statistical Analysis

Data on relative gene expression, enzyme activity, and plant growth were subjected to one-way analysis of variance (ANOVA) and the statistically significant differences of treated muskmelon plants and controls were analyzed by Student’s *t*-test and Duncan’s multiple range test and independent-sample *t*-test with threshold *p* < 0.05. Disease scales were subjected to ANOVA and analyzed by Kruskal–Wallis non-parametric statistical test. Statistically significant differences were analyzed by Mann–Whitney U test with threshold *p* < 0.01.

## 3. Results

### 3.1. Effect of Biotic Stress by T. asperelloides PSU-P1 on Gene Expression

**Figure 1:**
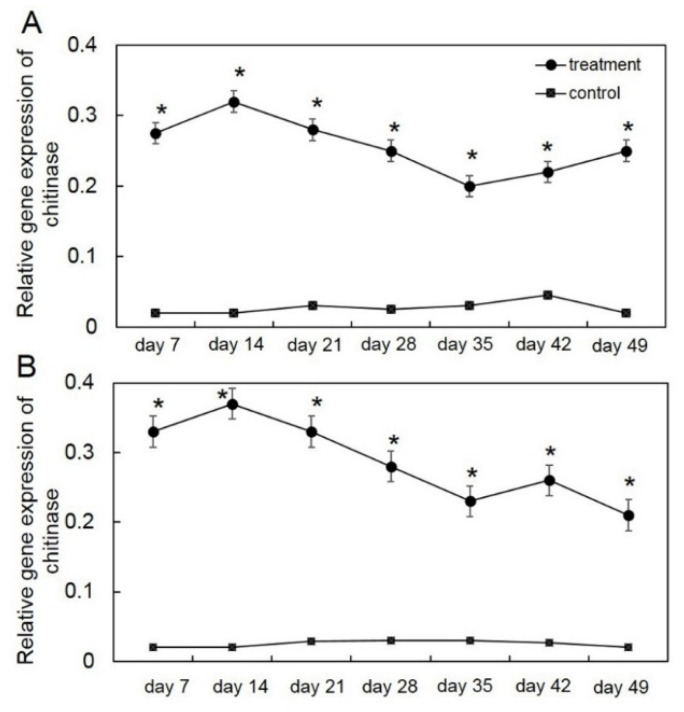
Relative gene expression of chitinase in the first crop (**A**) and second crop (**B**) through reverse transcription quantitative PCR (RT-qPCR) in *Trichoderma* *asperelloides* PSU-P1-treated muskmelon plants and control plants. * Indicates a significant difference between treatment and control according to Student’s *t*-test with *p* < 0.05.

**Figure 2:**
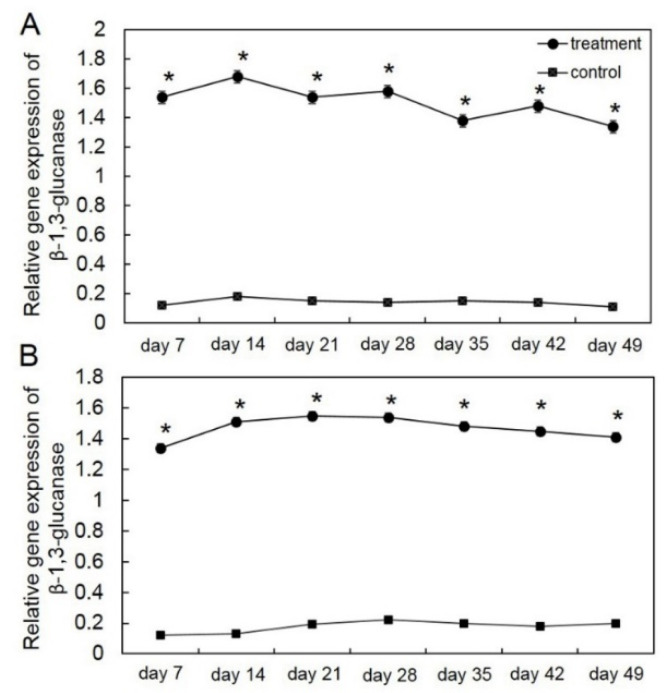
Relative gene expression of β-1,3-glucanase in the first crop (**A**) and second crop (**B**) through reverse transcription quantitative PCR (RT-qPCR) in *Trichoderma* *asperelloides* PSU-P1-treated muskmelon plants and control plants. * Indicates a significant difference between treatment and control according to Student’s *t*-test with *p* < 0.05.

To examine the effect of biotic stress by *T. asperelloides* PSU-P1 on inducing the defense response in muskmelon, the expression of *chi* and *glu* genes was analyzed by RT-qPCR. The expression levels of *chi* and *glu* genes in *T. asperelloides* PSU-P1 were 7–10-fold higher than those of the control (Figure 1 and Figure 2). In *T. asperelloides* PSU-P1-treated muskmelon plants, the expression levels of *chi* were 0.2–0.32 for the 1st crop and 0.21–0.37 for the 2nd crop (Figure 1), while the expression levels of *glu* were 1.34–1.68 for the 1st crop and 1.34–1.55 for the 2nd crop (Figure 2); both these expression patterns were significantly higher than the levels in the control.

### 3.2. Application of T. asperelloides PSU-P1 Elevated Enzyme Activity in Muskmelon

**Figure 3:**
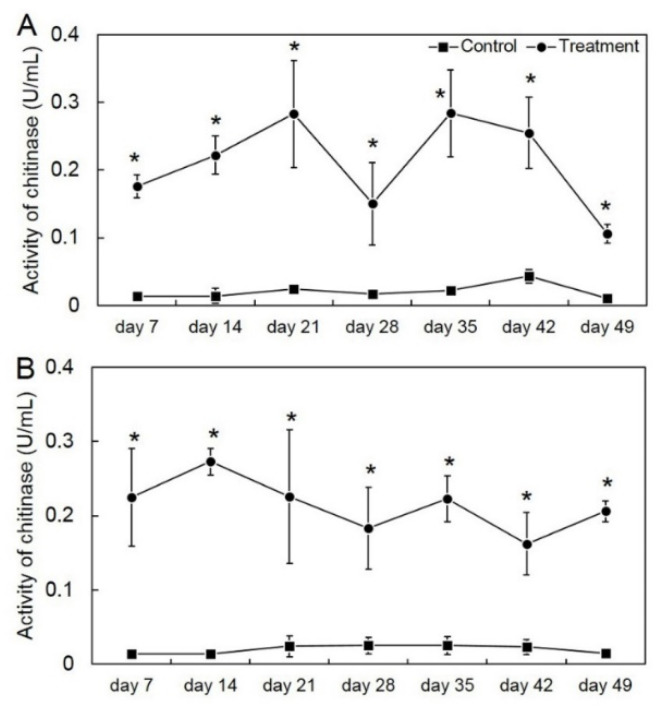
Activity of chitinase in the first crop (**A**) and second crop (**B**) through enzyme assay in *Trichoderma* *asperelloides* PSU-P1-treated muskmelon and control plants. * Indicates a significant difference between treatment and control according to Student’s *t*-test with *p* < 0.05.

**Figure 4:**
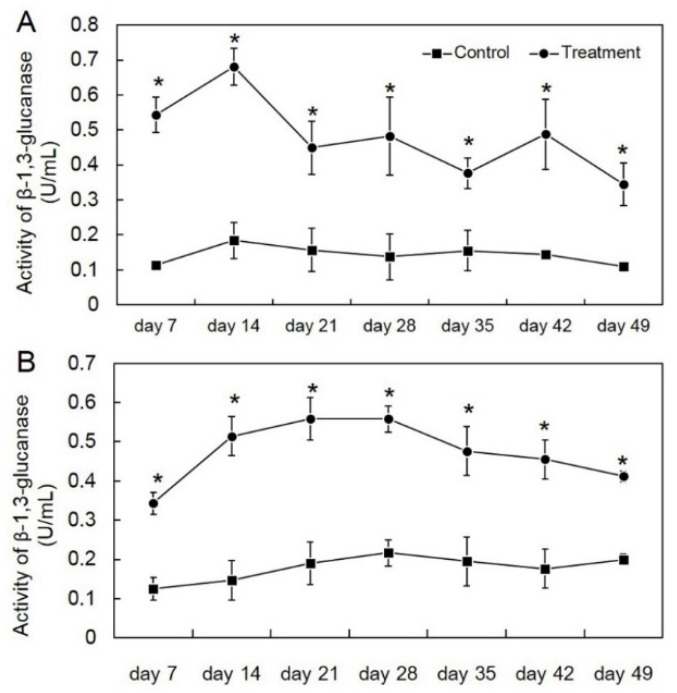
Activity of β-1,3-glucanase in the first crop (**A**) and second crop (**B**) through enzyme assay in *Trichoderma* *asperelloides* PSU-P1-treated muskmelon and control plants. * Indicates a significant difference between treatment and control according to Student’s *t*-test with *p* < 0.05.

We tested the effect of *T. asperelloides* PSU-P1 on CWDE activity in muskmelon plants from the seedling stage through to the harvesting stage. Inoculation of *T. asperelloides* PSU-P1 caused a high activity of CWDEs in this study. For the first crop, all *T. asperelloides* PSU-P1-treated muskmelon plants showed a significantly higher activity of chitinase and β-1,3-glucanase than those of the control (untreated muskmelon plants). The activities of chitinase and β-1,3-glucanase were 0.150–0.284 and 0.345–0.681 U/mL, respectively (Figure 3). For the second crop, the results were similar; the activities of chitinase and β-1,3-glucanase were 0.162–0.273 and 0.343–0.559 U/mL, respectively (Figure 4).

### 3.3. Effect of Crude Metabolites on Fungal Morphology

**Figure 5:**
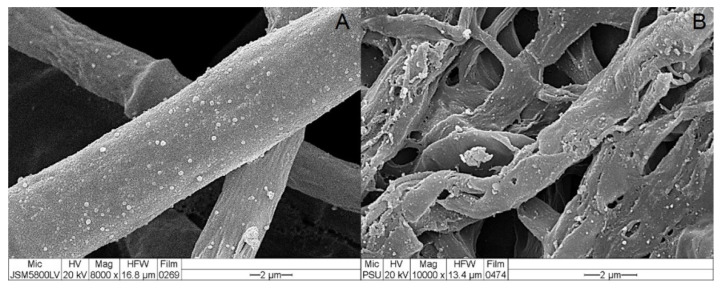
Effect of crude metabolite of *Trichoderma asperelloides* PSU-P1 treated muskmelon: (**A**) *Stagonosporopsis cucurbitacearum* hyphae treated with PDB (control), and (**B**) *S. cucurbitacearum* hyphae treated with a crude metabolite of *T. asperelloides* PSU-P1 observed by scanning electron microscope (SEM).

Crude metabolites extracted from *T. asperelloides* PSU-P1-treated muskmelon plants may contain CWDEs, which can cause morphology change, observed by wilting and lysis of *S. cucurbitacearum* hyphae. SEM micrographs showed wilted and lysed fungal morphology of *S. cucurbitacearum* incubated with *T. asperelloides* PSU-P1-treated muskmelon metabolites (Figure 5). The fungal morphology of *S. cucurbitacearum* incubated with untreated control metabolites remained healthy (Figure 5).

### 3.4. Effect of T. asperelloides PSU-P1 on Plant Growth and Postharvest Quality of Muskmelon Fruit

To test the effect of *T. asperelloides* PSU-P1 on the growth and yield of muskmelon, an experiment in a polyhouse was conducted for two crops. After treatment with the spore suspension of *T. asperelloides* PSU-P1 for about 2 months, stem diameter and shoot length were not significantly different between control and *T. asperelloides* PSU-P1-treated muskmelon plants in both crops (Table 2). Stem fresh weight, fruit weight, and fruit width in *T. asperelloides* PSU-P1-treated muskmelon plants were significantly higher than those of the control in both crops (Table 2). To investigate the quality of muskmelon fruit, fruit sweetness and fruit firmness were measured, and the results displayed no significant difference between treatment and control for both crops (Table 3).

### 3.5. Trichoderma asperelloides PSU-P1 Reduces Disease Severity in Field Crop

**Figure 6:**
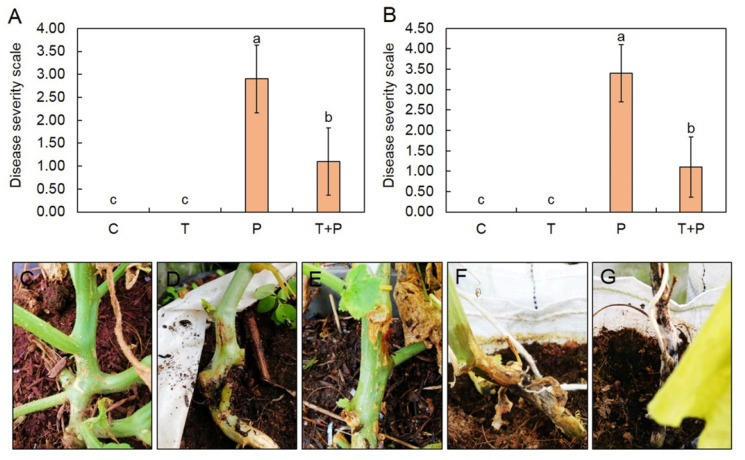
Qualitative disease severity scale of gummy stem blight in the first crop (**A**) and second crop (**B**). Representative images showing plants with qualitative severity scale ratings of: 0 (**C**), 1 (**D**), 2 (**E**), 3 (**F**), and 4 (**G**). T = *T. asperelloides* PSU-P1; P = *S. cucurbitacearum;* T + P = *T. asperelloides* PSU-P1 and *S. cucurbitacearum*. Different letters indicate statistically significant differences among treatments (*p* < 0.01) using Mann–Whitney U test.

We tested the effect of *T. asperelloides* PSU-P1 on the reduction of gummy stem blight in field trials. Treatment with *T. asperelloides* PSU-P1 reduced the qualitative disease severity scale caused by *S. cucurbitacearum* in both crops. For the first crop, the disease severity scale of *T. asperelloides* PSU-P1-treated muskmelon plants was 1.10 ± 0.74, which was significantly lower than that of the control (pathogen alone), 2.90 ± 0.74 (Figure 6). Similar results were observed for the second crop. *T. asperelloides* PSU-P1-treated muskmelon plants exhibited a disease severity scale of 1.10 ± 0.74, significantly lower than that of the control (pathogen alone), 3.40 ± 0.70 (Figure 6).

## 4. Discussion

In this study, we tested the effect of *T. asperelloides* PSU-P1 in terms of the response of PR genes against gummy stem blight of muskmelon in field trials. The experiments were conducted on two crops, and we found that *T. asperelloides* PSU-P1 upregulated PR protein genes, resulting in the release of CWDEs in muskmelon plants, causing disease resistance. Furthermore, the application of *T. asperelloides* PSU-P1 reduced disease severity in muskmelon plants in both crops.

The ability of BCAs to act as biotic stresses in plants has been widely studied in several plant species. BCAs induce disease resistance by upregulating pathogenesis-related gene expression [24]. For instance, the BCAs *T. viride* and *Bacillus subtilis* upregulated chitinase gene expression against *Fusarium oxysporum* and *Rhizoctonia solani* in tomato plants [25]. The application of non-pathogenic *Fusarium* as a BCA upregulated chitinase and β-glucanase in common bean [26]. Our study is in agreement with those previously reported, and showed that application of *T. asperelloides* PSU-P1 to muskmelon plants upregulated the expression of chitinase and β-1,3-glucanase genes. The results from both crops also confirmed this phenomenon (Figure 1 and Figure 2). Therefore, our results suggest that the upregulation of PR protein genes is associated with disease resistance against gummy stem blight in muskmelon plants.

In this study, we tested the ability of *T. asperelloides* PSU-P1 as a BCA to elevate CWDEs in muskmelon plants. We found that application of *T. asperelloides* PSU-P1 caused high activity of chitinase and β-1,3-glucanase in both investigated crops. It is known that application of BCAs induces enzyme activity in plants. For instance, the application of a biocontrol agent induced the defense mechanism in coconut palms, resulting in reduced *Ganoderma* disease incidence in [27]. Application of *T. asperellum* MSST induced chitinase and β-1,3-glucanase activities in tomato plants [28]. Furthermore, Baiyee et al. [5] reported that *T. asperellum* T1 induced disease resistance against leaf spot disease on lettuce. Crude metabolites of BCA-treated plants include CWDEs, which can cause abnormal changes of fungal mycelia [5,29]. Our results showed that crude metabolites of *T. asperelloides* PSU-P1-treated muskmelon plants contained lytic enzymes, which was confirmed by SEM observation (Figure 5). Therefore, *T. asperelloides* PSU-P1 may induce and elevate CWDEs in muskmelon plants, which involves the suppression of fungal pathogens.

In order to reduce excess use of synthetic fungicides, *Trichoderma* species have been used as BCAs to control several plant species worldwide [30]. They have been used for controlling soilborne fungal pathogens [31,32] and airborne fungi [33,34]. The application of *T. longibrachiatum* and *T. asperelloides* increased plant biomass and reduced pathogen DNA in maize root [35]. Furthermore, a new species of *T. phayaoense* exhibited biological control activity against gummy stem blight and promoted plant growth in muskmelon in [36]. In our previous study, *T. asperelloides* PSU-P1 was screened because of its strong antifungal ability against *S*. *cucurbitacearum* [20] and was selected for use as a BCA in a field trial in this study. Our current results showed that the application of *T. asperelloides* PSU-P1 in both crops reduced the disease severity compared to control. This finding suggests that the application of *T. asperelloides* PSU-P1 as a BCA may be effective in managing gummy stem blight in muskmelon.

The aim of BCAs is not only to induce the defense response in plants, but also to promote plant growth in field trials. *Trichoderma* spp. promote soybean growth, with high phosphorus uptake [37]. The application of *Trichoderma* in foliar fertilizer increased shoot length and number of leaves and accelerated the appearance of shoots in black pepper [38]. The direct addition of *T. longibrachiatum* and *T. asperelloides* to the seeds showed yield improvement and increased the growth parameter and crop of maize compared with non-infected plants [35]. Our results are in agreement with those studies. The application of *T. asperelloides* PSU-P1 as a BCA promoted plant growth, increasing the fresh weight, fruit weight, and fruit width in muskmelon. The ability to promote plant growth by *Trichoderma* species may be due to the capacity to induce phytohormones in plants [39]. However, we did not determine the phytohormones in this study. Moreover, the application of *Trichoderma* species has been known to not only control plant diseases but also maintain postharvest quality in mango [40] and banana [41]. In this study, postharvest quality (sweetness and firmness) was not significantly different between *T. asperelloides* PSU-P1-treated and untreated muskmelon plants. This result suggests that the application of a BCA, *T. asperelloides* PSU-P1, did not cause a negative impact on the postharvest quality of muskmelon fruits.

## 5. Conclusions

Our current study revealed that a strong BCA (*T. asperelloides* PSU-P1) induced a defense response in muskmelon plants by the upregulation of *chi* and *glu* gene expression and elevating CWDEs (chitinase and β-1,3-glucanase). *T. asperelloides* PSU-P1 as a BCA could be used to manage gummy stem blight in field trials in both crops in this study. The use of *T. asperelloides* PSU-P1 increased the growth of muskmelon and maintained the postharvest quality of muskmelon fruit.

## Figures and Tables

**Table 1:** Specific primer pairs for the gene expression determinations with reverse transcription quantitative polymerase chain reaction (RT-qPCR).

Genes	Accession Number	Primer	Sequences (5′→3′)	Product Size (bp)
*chi*	AF241538	Chi-F	CGTGGACCAATGCAACTCAA	242
		Chi-R	ATTCCCTGTGCTGTCATCCA	
*glu*	AF459794	Glu-F	TGGAGAAGAATGGTGGAGGA	188
		Glu-R	GTCAGACATGGCGAACACAT	
*act*	AY859055	ACT-F	TGGTATGGAAGCTGCAGGAA	158
		ACT-R	GGGCTGTGATTTCCTTGCTC	

**Table 2:** Effect of *Trichoderma asperelloides* PSU-P1 on muskmelon growth.

Crop	Treatment	Stem Diam. (cm) ^a^	Shoot Length (cm)	Stem Fresh Weight (g)	Fruit Weight (g)	Fruit Width (cm)
1st	Control	0.94 ± 0.19	193 ± 26.52	519.70 ± 22.63	1036.60 ± 22.86	38.84 ± 2.76
	Treatment	0.98 ± 0.05	205.80 ± 30.71	589.44 ± 14.27 *	1274.40 ± 60.78 *	43.10 ± 3.84 *
2nd	Control	1.15 ± 0.05	215 ± 10.62	862.87 ± 22.17	1450.50 ± 52.73	45.11 ± 0.68
	Treatment	1.22 ± 0.12	211.50 ± 10.52	907.25 ± 29.78 *	1529.90 ± 21.98 *	48.35 ± 1.12 *

^a^ Values are means ± SDs with 10 replications, values with asterisks are significantly different according to independent sample *t*-test (* *p* < 0.05).

**Table 3:** Effect of *Trichoderma asperelloides* PSU-P1 on postharvest quality of muskmelon.

Crop	Treatment	Brix (°Brix) ^a^	Texture (bar)
1st	Control	14.18 ± 1.94	4.80 ± 0.40
	Treatment	14.60 ± 0.96	5.16 ± 0.79
2nd	Control	12.53 ± 0.86	5.40 ± 0.50
	Treatment	13.00 ± 0.89	5.25 ± 0.98

^a^ Values are means ± SD with 10 replications.

## Data Availability

Not applicable.
